# Unpacking Resilience: Exploring the Link Between Dissociative Responses and Psychological Resilience in War-Affected Palestinians

**DOI:** 10.1192/j.eurpsy.2026.11996

**Published:** 2026-07-31

**Authors:** H. K. Yacoub

**Affiliations:** Faculty of Medicine, Al-Quds University, Abu Dis, East Jerusalem, West Bank and Gaza

## Abstract

**Introduction:**

Peritraumatic dissociation (PD) involves disruption in awareness, memory, identity, and perception that happens during or right after exposure to a traumatic event. PD has been described in the literature as a predictor of PTSD and is usually seen as a negative or maladaptive response. However, the relationship between PD and psychological resilience is still unclear, especially among populations exposed to war-related trauma. Resilience is defined as the ability to recover or adapt well in the face of adversity and might play a role in shaping how people experience trauma. The interaction between dissociation and resilience has rarely been studied in war zones or under chronic traumatic conditions.

**Objectives:**

To investigate the relationship between peritraumatic dissociation and resilience among civilians exposed to the 2024 Gaza–Israel war.

**Methods:**

A cross-sectional survey was conducted between December 2024 and February 2025. The study included 623 participants from Gaza, the West Bank, and Israeli-controlled areas. Peritraumatic dissociation was measured using the Peritraumatic Dissociative Experiences Questionnaire (PDEQ), and resilience was measured using the 10-item Connor-Davidson Resilience Scale (CD-RISC-10). Data were analyzed using SPSS v26. Descriptive statistics were calculated, and Pearson correlation and multiple regression analyses were conducted to assess the relationships between PD, resilience, and other variables.

**Results:**

Participants showed moderate levels of PD (M = 28.31, SD = 8.44) and resilience (M = 32.38, SD = 8.92). A significant positive correlation was found between resilience and PD (r = 0.404, p < .001). Female participants had significantly higher PD scores (p < .001), and participants with higher education had significantly higher resilience scores (p = .007). A history of mental illness was associated with both higher PD and lower resilience.

**Table 1.** Mean Scores and Significant Associations Table 1. Long description.

Variable	Mean (SD)	Statistical Association	p-value
Peritraumatic Dissociation (PDEQ)	28.31 (8.44)	—	—
Resilience (CD-RISC-10)	32.38 (8.92)	Positively correlated with PD (r = 0.404)	< .001
Gender (Female)	—	Associated with higher PD scores	< .001
Education Level (Higher)	—	Associated with higher resilience scores	0.007
History of Mental Illness	—	Associated with higher PD and lower resilience	< .001

**Conclusions:**

This study found a significant positive correlation between peritraumatic dissociation and resilience. This was unexpected and challenges the common view that dissociation only reflects poor coping. In the context of war, dissociative reactions may happen even in people with strong resilience. These findings call for a better understanding of how resilience and dissociation interact, and for trauma-informed mental health care that recognizes this complexity in conflict zones.

**Disclosure of Interest:**

None Declared

## Long descriptions

### Table 1. Long description

The table consists of four columns: Variable, Mean (S D), Statistical Association, and p-value.

* Row 1: Peritraumatic Dissociation (P D E Q) has a Mean (S D) of 28.31 (8.44). No statistical association or p-value is listed.

* Row 2: Resilience (C D-R I S C-10) has a Mean (S D) of 32.38 (8.92). It is positively correlated with P D (r = 0.404) with a p-value less than .001.

* Row 3: Gender (Female) is associated with higher P D scores with a p-value less than .001.

* Row 4: Education Level (Higher) is associated with higher resilience scores with a p-value of 0.007.

* Row 5: History of Mental Illness is associated with higher P D and lower resilience with a p-value less than .001.

Navigate back to Table 1..

